# Survey of knowledge, and attitudes to storage practices preempting the occurrence of filamentous fungi and mycotoxins in some Ghanaian staple foods and processed products

**DOI:** 10.1038/s41598-023-35275-5

**Published:** 2023-05-29

**Authors:** Nii Korley Kortei, Sandra Badzi, Salifu Nanga, Michael Wiafe-Kwagyan, Denick Nii Kotey Amon, George Tawia Odamtten

**Affiliations:** 1 School of Allied Health Sciences, Department of Nutrition and Dietetics, University of Health and Allied Sciences, PMB 31, Ho, Ghana; 2 School of Basic and Biomedical Sciences, Department of Basic Sciences, University of Health and Allied Sciences, PMB 31, Ho, Ghana; 3 College of Basic and Applied Sciences, Department of Plant and Environmental Biology, University of Ghana, P. O. Box LG 55, Legon, Ghana

**Keywords:** Biochemistry, Environmental sciences, Natural hazards

## Abstract

Mycotoxigenic fungi can infect and produce potent mycotoxins in foodstuffs prior to harvest, during harvest (field fungi), and in storage after harvest (storage fungi), which when ingested, can result in adverse health effects. This study was aimed at assessing the knowledge, attitudes, and practices adopted by the Ghanaian populace to help mitigate the occurrence of molds and mycotoxins in foods. A cross-sectional survey involving a structured questionnaire was conducted with 642 respondents from twelve regions of Ghana. Descriptive statistics and analyses of variance were calculated. Correct Classification Rate (CCR) was measured to assess the utility of a logistic regression model. The results of the study showed that the majority of 299 (46.6%) of the respondents were between the ages of 18–25. Age and educational level were related to knowledge about the occurrence of fungi and mycotoxins in foods (p < 0.05). More than half the respondents, 50% indicated that they knew of aflatoxins as a major mycotoxin present in food. Higher education directly influenced on the knowledge of mycotoxicosis and the management of stored food to present intoxication by fungal metabolites. 502 (32.9%) knew that consuming foods with toxins could cause stomach aches. The most commonly consumed food commodity despite the presence of visible growth of fungi was bread (35.3%). The average KAP score for knowledge showed that, out of 100%, there was adequate knowledge (63.8%) among the members of the Ghanaian populace. Favorable environmental conditions of high humidity (> 85% ERH) and temperature (> 28–32 °C) enhance the proliferation of fungi in most foods and the attendant production of mycotoxins such as aflatoxins, ochratoxins, and fumonisins are associated with several severe human and animal health conditions; mycotoxicosis was associated with high fever, pain, vomiting, suppression of immunity, cancer, etc. when these foods are consumed on regular basis for a prolonged length of time. Future examination of the food items used for the School Feeding Programme in Ghana will offer opportunities to examine the risks of feeding youth with fungal-contaminated food preparations from providers.

## Introduction

Fungal contamination of foodstuffs and agricultural produce is a prevailing global problem, particularly in developing countries. The prevailing hot and humid tropical environmental climate conditions support the growth of mycobiota both in the field and in storage^1^. Unfortunately, the growth of fungi causes not only spoilage but depreciates food quality and reduces food security. The ubiquitous nature of fungi and their airborne spores enable them to thrive over a wide range of habitats including deserts, hyper-saline environments such as the sea, on rocky surfaces, etc. with varying climatic conditions of low and high temperatures^2^.

The species of fungi causing mycotoxicosis are mainly in the genera of *Aspergillus, Fusarium, *and* Penicillium,* although others have also been implicated in causing mycotoxicosis in man when he ingests food contaminated by fungal toxins. Indeed, some mycotoxins cause diseases such as cancer (aflatoxins), and some damage vital organs such as the brain and nervous system, liver, and kidney to mention but a few^3,4^. Of great concern to public health is the effect of mycotoxins on the fragile immune systems of adults and children alike, although infants, by virtue of their smaller body weight and less acid stomach, are more susceptible to mycotoxins than adults^5,6^.

Indigenous farmers in Africa have devised the use of local plant parts to reduce fungal and insect infestation in stored staple foods^7,8^. However, fungal toxins continue to take a toll on the health of the populace. It is presumed that the lack of knowledge of the conditions which predispose food to fungal intoxication is not well understood by both the farmers and the populace although the phenomenon has been demonstrated by scientists in the pertinent literature^9^.

It is well known that aflatoxins are potent hepatotoxic, teratotoxic, mutagenic, and carcinogenic mycotoxins produced by members of the *Aspergillus* section Flavi namely *A. flavus, A. parasiticus, A. nomius, A. pseudonomius, A. arachidicola, A. minisclerotigenes, A. ochraceoroseus, A. korhogoencis, A. pseudocaelatus, A. pseudotamarii,* and *A. bombycis*^10^. Aflatoxins B_1_, B_2_, G_1_, and G_2_ can occur in foods such as groundnuts, tree nuts, maize, rice, figs, dried spices, crude vegetable oils, cocoa beans, cocoa powder, chocolate, cotton seeds, copra etc.^11,12^. Aflatoxins M_1_ and M_2_ are found in milk and milk products. Human hepatic cancer and acute fatal diseases eg. Hepatitis have occurred in association with the consumption of heavy contaminated foods in Asia, africa and else where^13,14^,

Ochratoxins are the main mycotoxin with nephrotoxic effects and have been associated with Balkan Endemic Nephropathy and tumour development in the urinary tract^15–17^. Ochratoxin A (OTA) is a toxic secondary metabolite produced by several species of *Aspergillus* and *Penicillium* genera. For example, OTA is produced by *Aspergillus carbonarius, A. sclerotiorum, A. sulphureus, A. alliaceus, A. affinis, A. albertensis, A. welwitschive, A. cretensis, A. alutaceus* (= *A ochraceus*) and *A. niger* and species belonging to the *A. niger* aggregation^18,19^ in tropical zones^18,20–22^. The European Food Safety Authority^23^ affirmed that OTA is nephrotoxic in all animal species tested and exerts immunotoxic, neurotoxic, and teratogenic effects at high dose levels. OTA has been detected in high contamination levels in maize, rye, coffee, cocoa powder, and chocolate, and other related foods in Africa^24–28^. *Penicillium verrucosum* and *P. nordicum* also grew well and produced OTA^18^.

Fumonisin is a group of fifteen (15) closely related mycotoxins produced by species of *Fusarium* i.e. *F. proliferatum, F. globosum, F. nygum, F. subglutinans, F. verticillioides* = (*F. moniliforme*) all included in the *Gibberella fujikuroi* species complex^29^. *F. verticillioides* is important in veterinary medicine as a cause of porcine pulmonary edema and equine leucoencephalomalacia and oesophageal cancer in humans. Several cogenes of fumonisins A_1_, A_2_, B_2_, B_3_ and B_4_ as well as P are known^29^. Studies have also shown that some strains of *Aspergillus niger, A. welwitschiae* as well as *Alternaria alternata* and *Fusarium oxysporum* also produce fumonisins. Fumonisins have been shown to occur in barley, wheat, sorghum, rice, millet and corn products^29^. The most common syndromes associated with fumonisins are leucoencephalomalacia in horses, pulmonary oedema and hydrothorax in pigs, and hepatotoxicity, carcinogenicity and oesophageal cancer in humans^9,30^.

Zearalenone, ZEA, previously known as F2-toxin in a resorcyclic acid lactone produced by *Fusarium graminearum, F. colmorum, F. crookwellense*, and *F. equiseti*^31,32^. Another mycotoxin of health importance is Vomitoxin which is also known as deoxynivalenol (DON) and is a type B trichothecene, an epoxy sesqueterpenoid. It occurs predominantly in grains, e.g. wheat, maize, barley, rye, oat, and less so in rice, sorghum and triticale^33^. It is a secondary metabolite of *Fusarium culmorum and F. graminearum*.

Patulin is isolated from several species belonging to the genera *Aspergillus, Penicillium, Paecilomyces,* and *Byssochlamys*. Among the *Aspergillus* species, the number of patulin producing species is limited to three of the *Clavali* group, namely, *A. clavatus, A. giganteus*, and *A. longivestica*^34^. Within the genus *Penicillium,* patulin-producing species are *P. carneum, P. glavigeum, P. concentricum, P. coprobium, P. dipodomyicola, P. expansum, P. patulum, Psclerotigenum* and *P. valpinum*^35^. In the case of *Paecilomyces* and *Byssochlamys*, *B. nivea* and *B. saturanus* produce patulin but *B. fulva* does not^36,37^. Currently, the list of mycotoxins levels in food is regulated in many countries including European Community and Africa are aflatoxins, ochratoxin A, zearalenone, fumonisins and trichothecene^36^.

Food safety is a vital gauge or criterion for enhanced food security. In sub–Saharan Africa where major food losses occur, health challenges and even human fatalities have arisen from eating contaminated key staple foods by fungal pathogens in the field (field fungi) and in storage (storage fungi)^38–40^.

The most effective way to prevent mycotoxin formation in stored staple food from the farm gate through the food value chain is to implement Good Agricultural Practices (GAP), Good Manufacturing Practices (GMP), and Hazard Analysis of the Produce at the Critical Control Point (HACCP) (13,022,000; 2005). Post-harvest practices (drying, sorting, removal of broken and mouldy ones, keeping perishable staples in well-ventilated storage houses etc.) could also help in reducing food quality losses not excepting preharvest practices such as crop rotation, row planting, etc.^6,41^.

In 2011, The Food and Agriculture Organisation, (FAO) funded a Continental Programme on Post-Harvest Losses (PHL) Reduction in Sub-Saharan Africa, including Ghana. The FAO^42^ PHL report covered staple food commodities (processed and unprocessed), e.g. maize, sorghum, millet, cowpea, rice, plantain, cassava, cocoyam, yam, fruits (mango, orange, pineapple, tangerine) oil palm, tomatoes, okra, groundnut, bambara groundnut, etc. poultry and poultry products, Livestock (cattle, sheep, goats, guinea fowls, fish (marine and artisanal etc.) and made recommendations to increase food security along the food value chain in Ghana. FAO made a recommendation for improving food safety and security.

Prior to the FAO^42^ PHL report, and thereafter there have been over 41 fungal species identified belonging to more than 20 genera recorded as field and storage contaminants of staple foods, spice and spice products, grains and grain products, pulse and beans in Ghana^43^. These fungal contaminants included species of the aforementioned mycotoxin-producing genera: *Aspergillus, Alternaria, Curvularia, Candida, Fusarium, Monilia, Geotrichum, Mucor, Neurospora, Oidiodendron, Paecilomyces, Penicillium, Pullularia, Rhizoctonia, Rhizopus, Syncephalastrum, Torula, Trichothecium, Saccharomyces* and *Trichoderma*^44–55^ etc. *Aspergillus* species (*A. alutaceus* = *A. ochraceus, A. candidus, A. fumigatus, A. glaucus, A. niger, A. sulphureus, A. terreus, A. amstelodami, A. ustus, A. nidulans, A. tamarii, A. flavus*) predominated over other species encountered followed by *Penicillium* (*P. digitatum, P. expansum, P. verrocusum, P. oxalicum, P. frequentans, P. purpurogenum, P. urticae*) *Paecilomyces* (*P. puntonii, P. varioti*); *Rhizopus* (*R. oligosporus, R. oryzae, R. stolonifer*); *Fusarium* (*F. verticillioides* = *F. moniliforme, F. nivale, F. oxysporum*), *Alternaria alternata*, *Curvularia lunata, Geotrichum albidium, Neurospora sitophila,* etc.^56^.

When potential mycotoxin-producing species are predisposed to conducive environmental conditions such as in the tropics, risk factors triggering mycotoxigenesis are induced into action, especially when farmers and the general population are not abreast with the risk factors because of the paucity of information.

The pertinent literature on mycotoxins found in Ghanaian staple foods, spices, cereals, peanuts, and processed grain and peanut products is replete with examples of mycotoxin contamination of foods. Table 1 summarizes mycotoxins detected in Ghana’s staple foods, food products, spices, and seasoning products. These findings underscore the view that there is a paucity of information on the application of techniques to curtail toxin formation in our foods. The prerequisites for extending the shelf life and quality of agricultural produce are not well understood by the populace and farm handlers of stored agricultural produce. To the best of our knowledge, there is hardly any published report on the knowledge, handling of stored foods, attitude to the consumption of mycotoxin-contaminated foods as well as the pre-and post-harvest handling of food to preclude proliferation and in vivo mycotoxin formation in stored agricultural produce in Ghana.

**Table 1:** Some mycotoxins detected in Ghanaian staple foodstuffs, spices, and spice mixtures kept under normal tropic ambient conditions.

Food products	Mycotoxin detected	Fungus	References
Maize grains (whole)	Aflatoxins	*Aspergillus flavus*	^57–59^
	Ochratoxins	*Aspergillus carbonarius, Penicillium verucosum*	
Maize powder	Aflatoxins	*A. flavus*	^12,60,61^
Fermented maize	Fumonisins	*Fusarium verticillioides*	^62–64^
	Aflatoxins	*A. flavus*	
Kenkey	Aflatoxins	*A. flavus*	^60^
Ice kenkey	Aflatoxins	*A*. *flavus*	^65^
Weanimix cereals	Fumonisins	*Fusarium verticillioides*	^66^
	Aflatoxin	*A. flavus*	^67^
Maize-groundnut mix	Aflatoxins	*A. flavus*	^68^
Dry cassava products	Aflatoxins	*A. flavus*	^47^
Kokonte	Aflatoxins	*A. flavus*	^47^
Cassava flour	Sterigmatocystin	*A. versicolor*	^47^
	Tentazonic acid	*Alternaria spp.*	
	Cyclopiazonic acid	*Aspergillus spp., Penicillium spp.*	
	Penicillinic acid		
	Patulin		
Cereals	Aflatoxins	*A.flavus*	^69,70^
Peanut/groundnuts	Aflatoxins	*A.flavus*	^71–74^
Raw cow milk	Aflatoxin M1	*A. flavus*	^75–77^
‘Brukina’ (fermented millet based milk beverage)	Aflatoxin M1	*A. flavus*	^78^
‘Wagashie’ (traditional soft cottage cheese)	Aflatoxin M1	*A. flavus*	^79^
Aqueous extract of fruit of pepper, okra, tomato	Aflatoxins	*A. flavus*	^80^
Millet	Aflatoxins		^81^
Seasoning powder spices	N.D	*A. flavus, Penicillium spp*	^43^
Spice and spice products	N.D	*A. flavus, Penicillium spp*	^45^
Kebab spice mix	Aflatoxins	Miscellaneous including* A. flavus*	^46^
Spices and herbs	Aflatoxins	Miscellaneous including *A.flavus* and *Fusarium sp*.	^82,83^
Fruits and Vegetables	Aflatoxins	*A. flavus*	^84^
Nuts and oils	Aflatoxins	*A. flavus*	^84^
Plantains	Fumonisins	*Fusarium sp.*	^85^
	Ochratoxins	*Penicillium sp.*	^85^
Cocoa beans	Aflatoxins	*A. flavus*	^86^
	Ochratoxins	*A. carbonarius*	^86^
Animal feed	Aflatoxins	*A. flavus*	^84^

## Methodology

### Study design

This study was both qualitative and quantitative research. A prospective cross-sectional study design was used to collate information from food vendors and members of the Ghanaian populace and their basic knowledge, attitudes, and practices to mitigate infection of fungi in foods using a structured online questionnaire.

### Study site

**Figure 1:**
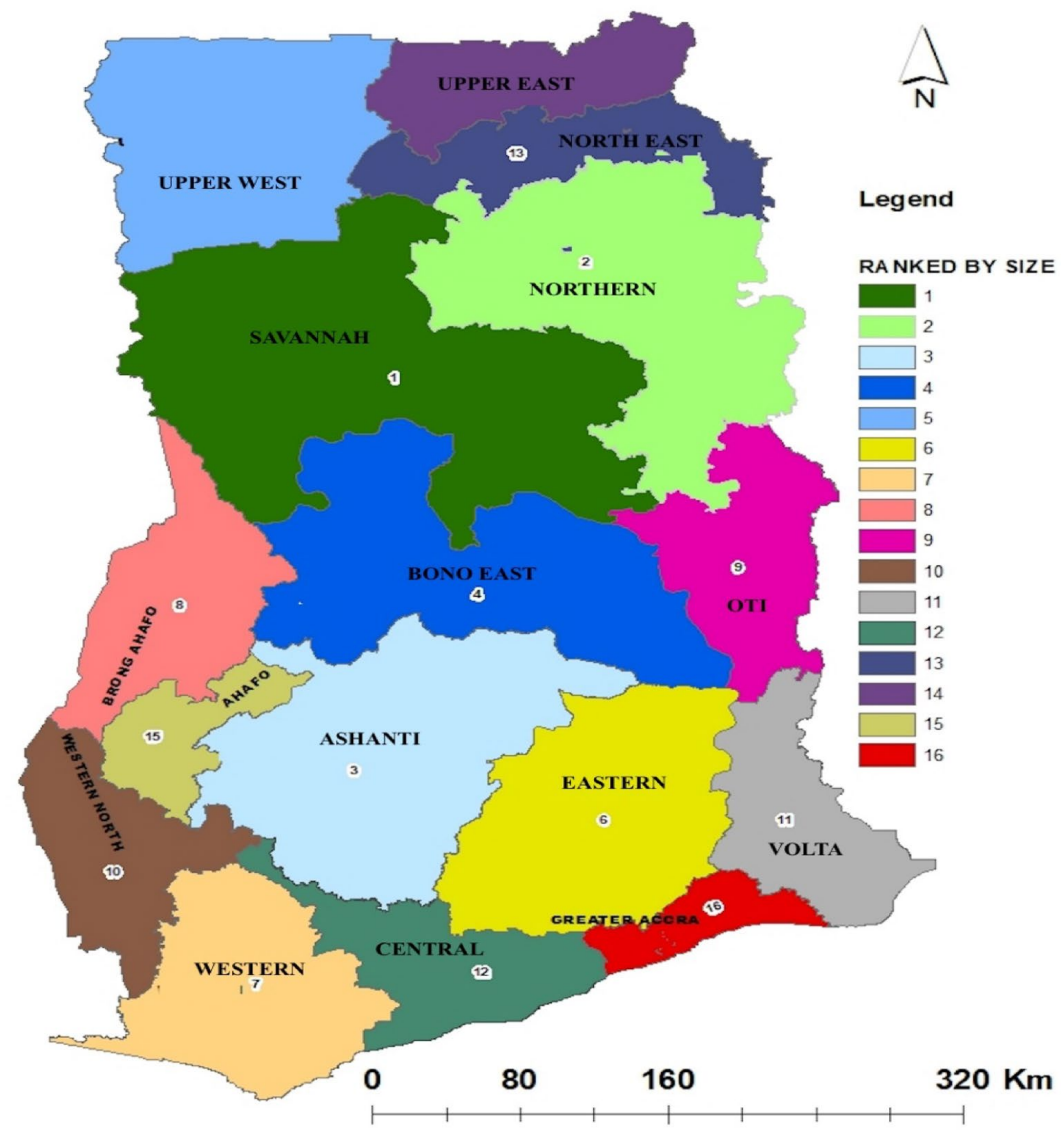
Map of Ghana showing the sixteen regions where sampling of views was carried out to assess the information required. Source: https://upload.wikimedia.org/wikipedia/commons/e/e7/NEW_GHANA_REGIONS.jpg.

The study was carried out across the country. Ghana is on the Gulf of Guinea, West Africa, where it covers about 23,884,245 ha of land and water and is positioned between latitudes 4°N and 11°N and longitudes 4° W and 2°E. Sixteen (16) regions and 216 districts constitute the country and are well defined by five broadly characterized agro – ecological zones, namely, Coastal Savannah, Evergreen Deciduous Forest, Transitional, and Savannah (Fig. 1). The population of Ghana currently stands at 31,000,062 people according to the data of Ghana Statistical Service^87^.

#### Desk study

A desk study was carried out to collate available information (on the internet) in Ghana on the occurrence and detection of some principal mycotoxins found in food. This is presented in Table 1. This spans the period 1986–2022.

Key—Population of regions.

**Table:** 

1 = 653,266	2 = 2479	3 = 4,780,380	4 = 1,208,699
5 = 868,479	6 = 2,633,154	7 = 3,093,000	8 = 2,310,983
9 = 742,664	10 = 711,435	11 = 2,118,252	12 = 2,201,863
13 = 658,903	14 = 1,046,545	15 = 564,536	16 = 5,455,692

### Inclusion criteria

Members of the Ghanaian populace who have basic knowledge in food safety.

### Exclusion criteria

Members of the Ghanaian populace who do not have basic knowledge in food safety.

### Determination of sample size

We adopted the prescribed formula of Yamane (1967) where:

$$ n\, = \,\frac{N}{{1\, + \,Ne^{2} }}. $$

In this formula, “*n”* represents the sample size to be calculated, while “*N*” is the relevant population. The value of “*e*‟ (standard error) depends on the required confidence level set by the researcher. If the confidence level is 0.95 percent, then the “e‟ value would be 0.05. In this study, a 95% confidence level was adopted. Our check revealed that the population of Ghana is about 31,000,000 which also represents food consumers in Ghana. Therefore, for *N* = 31,000,000, *e* = 5%, therefore n = 385.10 which approximates to 385. This implies that the minimum recommended size (number) is 385 respondents. We exceeded this and recruited 642 respondents nationwide.

### Data collection

An online questionnaire was designed for the national survey and divided into 4 sections: socio–demographic characteristics; basic knowledge of the respondent on fungal presence in foods; respondent practices on the control and management of fungi in foods; respondent in-depth knowledge and management of fungi in foods. In the socio–demographic section, respondents were asked to provide information on their age, sex, occupation, and residential region. The basic knowledge of respondents on the presence of fungi in food allowed respondents to portray their layman’s knowledge about the presence of fungi in foods. The respondent’s practice for the control and management of fungi in food gave a clue as to how they handled foods that have been contaminated with mycotoxins and how they manage the occurrence of fungi. On the question of attitude towards the knowledge and management of fungi in foods, we derived from the respondents their knowledge and management of fungi in food. After due consultation with respondents who were 18 years and above, the questionnaire was uploaded onto all social media platforms.

The online survey was conducted between April to June 2021. A reminder was sent to all potential respondents in July 2021. All returned responses were checked and verified to ensure proper completion. A total of 642 validated questionnaires were returned.

#### Fully completed survey forms

Fully completed surveys were deemed valid responses. English was the main language used to communicate in the interview and questionnaire, but where there was the need for a different language, help was provided by an interpreter. All our national COVID-19 protocols were strictly adhered to, i.e. ensuring the wearing of face masks, the use of sanitizers, washing of hands thoroughly, and socially distancing during the survey.

All methods were carried out in accordance with relevant guidelines and regulations.

### Ethics approval and consent to participate

Ethical clearance and permission were sought from the University of Health and Allied Sciences (UHAS) ethical clearance committee with Protocol Identification Number: UHAS-REC A.9^46^ 20–21. We confirm that informed consent was obtained from all subjects.

### Statistical analysis

Completeness and consistency of the data were checked and entered into Statistical Package for Social Sciences (IBM SPSS) data analysis software version 20 was employed to analyze the quantitative data obtained from the respondents. IBM SPSS was used to detect and remove inaccurate and incomplete data. Results were presented as descriptive statistics in tables, and graphs to facilitate interpretation. A threshold of significance was set at < 0.05 (p < 0.05).

The Chi-Square test of association was carried out to determine whether there was a significant association between categorical variables. The Mann–Whitney test was also used to test differences between two groups, while the Kruskal–Wallis test was used to test differences between more than two groups. The Conover-Iman pairwise comparison test was carried out on the categories within the variables.The statistical Hosmer–Lemeshow test, correct classification rate (CCR), and Area under the curve (AUC) were considered as the parameters for the goodness of fit test for the model. The Hosmer–Lemeshow test measured how poorly a model predicts an event of interest. Another useful measure to assess the utility of a logistic regression model is the correct classification rate (CCR). The area under the curve (AUC) is a measure of the ability of a classifier to distinguish between classes and is used as a summary of the ROC curve. Finally, Wilcoxon paired sample comparison test was used to test the familiarity of the respondents with yeasts and molds as well as mycotoxins likely to be found in the foods. Microsoft Excel version 2013 was used to plot the graphs.

## Results

The results of the desk study on the incidence and detection of mycotoxins in foods in Ghana are summarized in Table 1. The products include staple foods, dehydrated and processed products, etc. Mycotoxins detected were aflatoxins, fumonisins, sterigmatocystin, tenuazonic acid, patulin, and penicillanic acid (Table 1). Fungal genera producing these toxins were predominantly in the genus *Aspergillus, Penicillium,* and *Fusarium* (Table 1).

### Demographic data

The demographical data is summarized in Table 2. The majority of the respondents were between the ages of 18–25 years (46.6%); older people of 45 years and above formed the minority (1.9%). The respondents were predominated by males (52.3%) and by females (47.7%). The married constituted 69.3% and the remaining 30.7% were single. The majority of the respondents (80.4%) were either studying in tertiary education or had completed it, and only 3.0% had informal education. Interestingly, 44.5% of the total respondents have had a formal education; 52.5% were students and 3% were in informal education.

**Table 2:** Demographic characteristics of respondents.

Variables	Categories	Freq	%
Age	18–25	304	47.3%
	26–35	231	36.0%
	36–45	95	14.8%
	Above 45	12	1.9%
Gender	Female	306	47.7%
	Male	336	52.3%
Educational level	Basic education	3	0.5%
	Post-graduate education	92	14.3%
	Secondary education	31	4.8%
	Tertiary education	516	80.4%
Marital status	Married	197	30.7%
	Single	445	69.3%
Occupation	Formal	286	44.5%
	Informal	19	3.0%
	student	337	52.5%
Region of respondent	Ahafo region	7	1.1%
	Ashanti region	63	9.8%
	Bono east region	7	1.1%
	Bono region	14	2.2%
	Central region	32	5.0%
	Eastern region	64	10.0%
	Greater Accra	136	21.2%
	North east	5	0.8%
	Northern region	47	7.3%
	Oti region	8	1.2%
	Savannah region	12	1.9%
	Upper east region	26	4.0%
	Upper west region	30	4.7%
	Volta region	159	24.8%
	Western north region	5	0.8%
	Western region	27	4.2%

There was an inequitable return of questionnaires from the various regions. Most respondents resided in the Volta Region (24.8%) followed by Greater Accra (21.2%) and Eastern Region (10.0%) followed by Ashanti Region (9.8%). The remaining regions recorded less than 10% response with the least coming from North East (0.8%) and Western North (0.8%).

### Knowledge of the types of mycotoxins in foods

Respondents indicated their knowledge of the mycotoxins likely to occur in Ghanaian foods. There were multiple responses and a summary of the results is shown in Table 3. The majority of the respondents selected aflatoxins 453/843 (55.7%) followed by ochratoxin 137/843 (16.3%) and fumonisins 136/843 (16.1%). Zearalenone 54/843 (6.4%) was less known to the respondents. The remaining mycotoxins (patulin, trichothecene, ergot alkaloids, vomitoxin (deoxynivalenol)) scored 01–0.6% (Table 3). Interestingly, 0.4% and 0.1% of the respondents believed that yeasts and bacteria, respectively, also produce mycotoxins. Finally, 5.7% had no idea about mycotoxins in foods (Table 3).

**Table 3:** Multiple response analysis on the knowledge about types of mycotoxins in Ghana.

Mycotoxins	N	%	% of cases
Aflatoxins	453	53.7%	70.7%
Ochratoxin	137	16.3%	21.4%
Fumonisin	136	16.1%	21.2%
Zearalenone	54	6.4%	8.4%
Patulin	5	0.6%	0.8%
Neurotoxin	2	0.2%	0.3%
Trichothecenes	2	0.2%	0.3%
Ergot alkaloids	1	0.1%	0.2%
Vomitoxin (DON)	1	0.1%	0.2%
Yeast	3	0.4%	0.5%
Bacteria and fungi	1	0.1%	0.2%
Don’t know	48	5.7%	7.5%
Total	843	100%	131.5%

### Identification of food commodities predisposed to infection by moulds and yeasts

Moulds and yeasts are ubiquitous and can be resident preferentially in any nutrient-rich substrate for their growth^88^. In this survey, respondents identified fruits, vegetables, and fish (12.7–16.9%) as the most susceptible to infection; cereals, meat, root, and tuber crops also experienced mouldiness (12.8–14.6%) while bread was mentioned by only 2.1% of the respondents (Table 4). Peanuts (groundnuts) and other nuts were also prone to contamination (10.7%). Interestingly, respondents found kenkey less susceptible (0.3%), contrary to the general view of the populace.

**Table 4:** Multiple response analysis by panelists on food commodities or processed food susceptibility to fungal and yeasts contamination.

Food commodity	N	%	% of cases
Bread	43	2.1%	6.7%
Cereals	294	14.5%	45.9%
Meat	260	12.8%	40.6%
Root and tubers	296	14.6%	46.2%
Peanuts and other nuts	216	10.7%	33.8%
Vegetables	310	15.3%	48.4%
Fruits	343	16.9%	53.6%
Fish	257	12.7%	40.2%
Kenkey	7	0.3%	1.1%
Total	2026	100%	316.6%

### Multiple analysis of the response of respondents to the consumption of food despite identification of visible fungal growth

Table 5 summarises the results obtained from the survey using the questionnaire. Bread was the most consumed commodity (35.5%) despite visible signs of fungal contamination. This was followed by fish (9.9%), fruits (9.5%), meat (9.5%), and vegetables (9.0%). Only 0.2–0.3% of respondents ate infected root tubers and banku. Curiously, about 1.1% of the respondents avoided eating contaminated kenkey and 13.0% did not eat any of the listed foods found infected with fungi (Table 5).

**Table 5:** Multiple response analysis by the respondents of foods consumed in spite of visible fungi growth on the commodity.

Categories	N	%	% of cases
Bread	394	35.3%	61.8%
cereals	88	7.9%	13.8%
fruits	106	9.5%	16.6%
vegetables	100	9.0%	15.7%
Meat	106	9.5%	16.6%
Pea nuts and other nuts	48	4.3%	7.5%
None of the foods	145	13.0%	22.7%
Kenkey	12	1.1%	1.9%
Fish	111	9.9%	17.4%
Tubers	2	0.2%	0.3%
Banku	3	0.3%	0.5%
Total	1115	100%	174.9%

The respondents from the regions adduced several reasons for attempting to eat food visibly infected with fungi as shown in Table 5. For example, 55.6% of the respondents consumed food visibly infected with fungi. Some respondents (15.5%) also ate food with visible fungal contamination because they believed nothing fatal happened to them after eating and a further 3.8% of respondents purchased such contaminated food because they were cheaper on the market (Table 6). A greater percentage (6.5%) did not find any change in taste after the contaminated food was cooked for eating.

**Table 6:** Multiple response analysis by respondent’s attitude towards consuming foods showing visible fungal growth.

Categories	N	%	% of cases
Shortage of food	74	10.7%	15.0%
Scrape off, wash or cut infected portion	383	55.6%	77.5%
Nothing happened after eating	107	15.5%	21.7%
The taste of the food does not change on cooking	44	6.4%	8.9%
Do not believe they are harmful toxins	55	8.0%	11.1%
Contaminated foods are cheaper on the market	26	3.8%	5.3%
Total	689	100%	139.5%

### Knowledge of the adverse health effects on humans who consume contaminated foods intoxicated with mycotoxins

Table 7 is a summary of the results obtained. About one-third (32.9%) of the respondents believe that consumption of mycotoxin-contaminated foods can cause stomach upsets and aching. On the other hand, 30.8% attributed the incidence of diarrhea after toxin intoxication to the ingestion of contaminated food. Still others (15.5%) indicated that immunosuppression can be attributed to mycotoxins, while some 11.7% indicated that toxins can cause fever. The remaining 9.0% had indicated that mycotoxins can be carcinogenic in their effect on humans and animals alike.

### Knowledge and application of practices for the control and management of fungal contamination in stored foods

Although half of the respondents (50.7%) had seldom heard about fungi and their growth in foods, 34–38% either never heard about it and 5.3% had no clue about it (Table 8). Curiously, 77.9% of the respondents follow the traditionally prescribed storage conditions to preserve food. About 6.7–12.6% either discard visibly contaminated food or use only fresh food for cooking and consumption purposes (Table 8).

### Statistical relatedness of data obtained from the survey

Tables 2, 3, 4, 5, 6, 7, 9, 10, 11, 12 and 13 summarize the rest of the statistical analysis of the data obtained from the questionnaire.

**Table 7:** Multiple response analysis on knowledge of the adverse effects of mycotoxins on consumers.

Type of ailment	N cumulative response	%	% of cases
Stomach ache	502	32.9%	78.7%
Cancer	137	9.0%	21.5%
Immuno-suppression	236	15.5%	37.0%
Diarrhea	470	30.8%	73.7%
Fever	179	11.7%	28.1%
Total	1524	100%	238.9%

**Table 8:** Knowledge and application of storage management practices.

Questions on knowledge and answers application of management practice		Freq	N %
Do you hear often about fungi, yeast, or mold growth and its harmful effects?	Always	55	8.6%
	Frequently	190	29.5%
	Never	38	5.9%
	No idea	34	5.3%
	Seldom	326	50.7%
Can fungal growth in foods be prevented or controlled?	No	25	3.9%
	Yes	618	96.1%
How can fungi in foods be controlled or prevented?	Discarding foods with visible fungal growth	81	12.6%
	Ensuring clean environment	18	2.8%
	Proper storage of foods	501	77.9%
	Use of fresh food commodities in cooking	43	6.7%

**Table 9:** Differences in knowledge among demographic groups.

Variables	Mean	SD	Freq	%	P-value
Gender					
Female	4.376	0.898	306	47.7	0.3678
Male	4.452	0.948	336	52.3	
Age					
Below 18	3.800^**a**^	0.447	5	0.8	0.039*
18–25	4.321^**a**^	0.903	299	46.6	
26–35	4.498^**a,b**^	0.913	231	36	
36–45	4.526^**b**^	0.988	95	14.8	
Above 45	4.583^**a,b**^	1.084	12	1.9	
Educational level					
Basic education	4.667^**b**^	0.577	3	0.5	0.008*
Secondary education	3.839^**a**^	0.898	31	4.8	
Tertiary education	4.442^**b**^	0.911	516	80.4	
Post-graduate education	4.457^**b**^	0.965	92	14.3	
Marital status					
Married	4.447	0.922	197	30.7	0.2819
Single	4.402	0.926	445	69.3	
Occupation					
Formal	4.458	0.972	286	44.5	0.858
Informal	4.368	0.831	19	3.0	
Student	4.383	0.889	337	52.5	
Region					
Ahafo region	4.571	0.787	7	1.1	0.777
Ashanti region	4.460	0.820	63	9.8	
Bono east region	4.571	0.535	7	1.1	
Bono region	4.143	0.663	14	2.2	
Central region	4.500	1.016	32	5.0	
Eastern region	4.547	0.942	64	10.0	
Greater Accra	4.279	0.875	136	21.2	
North east	4.600	0.548	5	0.8	
Northern region	4.426	0.972	47	7.3	
Oti region	4.250	1.035	8	1.2	
Savannah region	4.250	1.215	12	1.9	
Upper east region	4.846	1.223	26	4.9	
Upper west region	4.367	0.928	30	4.7	
Volta region	4.415	0.923	159	24.8	
Western north region	4.200	0.837	5	0.8	
Western region	4.407	0.971	27	4.2	

Kruskal Wallis test was used to whether significant differences existed between groups.

Significant differences existed between the categories under age and educational level.

The Conover-Iman pairwise comparison test was carried out on the categories within the variables.

Significant values are in bold.

Means with same superscript letters are not significantly different (p>0.05).

Means with superscript asterisk (*) are significantly different (p<0.05).

**Table 10:** Hosmer–Lemeshow test measuring the odds of basic knowledge of fungi presence in different age groups.

Source	P-value	OR	OR L (95%)	OR U (95%)
Intercept	0.519			
Age group				
18–25				
26–35	0.032	2.526	1.083	5.890
36–45	0.020	4.194	1.256	14.007
Above 45	0.026	4.444	0.539	36.620
Below 18	0.001	0.999	0.103	9.733
Gender				
Female				
Male	0.620	0.878	0.524	1.470
Educational level				
Basic education				
Post-graduate education	0.817	0.635	0.014	29.614
Secondary education	0.418	0.214	0.005	8.984
Tertiary education	0.976	0.945	0.022	41.233
Marital status				
Married				
Single	0.112	1.952	0.856	4.453
Occupation				
Formal				
Informal	0.723	1.307	0.299	5.718
Student	0.023	2.172	1.110	4.249
Region				
Ahafo region				
Ashanti region	0.836	0.710	0.028	18.029
Bono east region	0.794	0.562	0.007	43.034
Bono region	0.445	0.263	0.009	8.072
Central region	0.962	1.089	0.033	36.504
Eastern region	0.758	0.602	0.024	15.279
Greater Accra	0.684	0.517	0.022	12.337
North east	0.845	0.643	0.008	53.595
Northern region	0.583	0.404	0.016	10.289
Oti region	0.410	0.226	0.007	7.737
Savannah region	0.713	0.510	0.014	18.573
Upper east region	0.602	0.413	0.015	11.510
Upper west region	0.492	0.317	0.012	8.426
Volta region	0.615	0.443	0.019	10.569
Western north region	0.409	0.206	0.005	8.805
Western region	0.837	0.701	0.024	20.547
Goodness fit test				
Hosmer–Lemeshow test	P-value = 0.767			
CCR (%)	90.03%			
AUC	66.60%			

*OR* odds ratio, *ORL* odds ratio lower boundary, *ORU* odds ratio upper boundary.

**Table 11:** Average knowledge, attitude and practice (KAP) score obtained from respondents.

Average KAP score (out of a possible 7)	Average KAP score (out of a possible 100)
4.42 ± 0.92	63.08 ± 13.21%

**Table 12:** Respondents familiarity with the terms mold, yeasts, and mycotoxins.

Familiarity with molds, yeasts, and mycotoxins using Wilcoxon paired sample comparison test						
Statements	Responses % (n)			Mean	SD	P-value
	1*	2*	3*			
How familiar are you with these terms; molds and yeasts	2.8 (18)	51.2 (329)	46.0 (295)	2.431	0.549	< 0.0001*
How familiar are you with the term ‘Mycotoxins’	24.8 (159)	49.1 (315)	26.2 (168)	2.014	0.714	
Overall mean score (n = 632)				**2.223**	**0.554**	

1* no idea, 2* quite familiar, 3* very familiar.

Significant values are in bold.

**Table 13:** Respondent practices for the control and management of fungi in food.

Variable	Knowledge score category				
		Score < 50%	Score ≥ 50%	Total %	P-value
Do you consume food showing visible fungi growth?	No	35	327	56.4	0.013*
	Sometimes	20	211	36.0	
	Yes	11	38	7.6	
Do you check for the presence of molds and fungi on foods before consumption?	No	17	104	18.8	0.13
	Yes	49	472	81.2	

Scores < 50% indicate inadequate knowledge and Scores ≥ 50% indicate adequate knowledge.

*Significant at 5%.

Significant values are in bold.

Results in Table 8 show that age was directly related to knowledge about the occurrence of fungi and mycotoxins in foods (*p* < 0.05); educational level also was positively related to knowledge about the occurrence of fungi and mycotoxins in foods (*p* < 0.05). All other demographic characteristics investigated (marital status, occupation, regional affiliation) were found to have no significant (p > 0.05) influence on the knowledge status of the respondents.

From Table 9, the same alphabet superscript assigned to categories denotes insignificant differences between groups. Different alphabets denote significant differences between groups.

Knowledge among age groups below 18 and 18–25 is significantly different from the age group 36–45.

Knowledge among those with secondary education is significantly different from those with basic, tertiary, and postgraduate education.

The Hosmer–Lemeshow test which is a statistical test for the logistic regression model is used frequently as a risk prediction model. Results showed that the model is a good fit (P-Value > 0.767). The CCR of 90.03% and the AUC of 66.60% also confirm that the model is a good fit ( Table 10).

With knowledge status as the dependent variable, the independent variables in the model were statistically insignificant except for the age group variable. Age groups above 45 years have increased odds of basic knowledge of fungi presence than the age group 36–45. Age groups 36–45 also have increased odds of basic knowledge of fungi presence than the age group 25–35. Age groups 25–35 also have increased odds of basic knowledge of fungi presence than the age group below 18 years. The age group 18–25 was used as the reference group.

In the Table 11, the average KAP score for knowledge indicated that, out of a total of 100%, there was adequate knowledge (63.8%) among the members of the Ghanaian populace. As to how familiar the respondents were with the terms mold and yeast, a mean score of 2.431 was obtained; how familiar the respondents were with the term mycotoxin recorded a mean score of 2.014 which is lower than the average mean of the knowledge of respondents. The results show that the familiarity of the respondents with the terms; molds, yeast, and mycotoxins were directly related to their knowledge about the occurrence of mycotoxins in foods (*p* < 0.05) (Table 12).

The Chi-square test of association was conducted to ascertain whether a statistical relationship existed between the practices for the control and management of fungi in food and their knowledge of the occurrence of fungi in foods. In terms of the practice of management and control of fungi in foods, respondents who did not consume food showing visible fungi growth constituted 56.4%; respondents who consumed food with visible fungi growth constituted with them was 36.0%. Results in Table 13 show that the consumption of food showing visible fungal growth is significantly (p < 0.05) related to the knowledge about the occurrence of fungi in foods. Respondents who inspected food for the presence of fungi on foods before consumption was 81.2%, while respondents who did not check for the presence of molds and fungi on foods before consumption constituted 18.8%. The results showed that it is not related to the knowledge of the occurrence of fungi in foods (p > 0.05).

## Discussion

Mycotoxin contamination of foods and processed food products is a universal problem that denies the grains from crop harvest the full benefits for use in food security. No country is exempt from fungal intoxication in food. The results of the desktop study showed a whole array of foodstuffs contaminated by potent mycotoxins of health importance and belonging to the genera *Aspergillus, Penicillium,* and *Fusarium* (Table 1). Local research into the occurrence of mycotoxins in Ghana is encouraging but a lot more needs to be done particularly in the area of damage to human health and its long-term effects on longevity. The pertinent literature in West Africa is replete with studies on the incidence of mycotoxins in staple edible foods and spices. Mycotoxins detected include aflatoxins, fumonisins, ochratoxins, penicillinic acid, patulin, vomitoxin (deoxynivalenol), zearalenone, trichothecenes^28,38,89–96^. The same is true for East and Central Africa^6,38,97–100^. Extensive studies on mycotoxins in staples, edible foods, and spices have also been carried out in Southern Africa^101^ and Egypt^102,103^. What needs to be underscored, is the education of the population to identify conditions of storage that predispose stored foods to mycotoxin contamination and the need to avoid the use of mouldy foods for animal and human consumption.

Results from the current study show that the majority of the respondents in the study were between the youthful ages of 18–25 yrs and had adequate knowledge about the contamination of foods by fungi. This agrees with the findings of^104^ who found the knowledge of natural toxins in foods by students of ages between 21 and 25 yrs in tertiary institutions in Ghana. This trend could be attributed to the larger volume of information on the internet available to the computer-literate youth on their mobile phones, laptops, and other gadgets. Another source of information on food intoxication could come from occasional educative FM radio, TV programs, and videos that they could access from their phones out of curiosity.

Our present result contrasts the findings of^97^ in Kenya. They showed that respondents above 35yrs were more knowledgeable about fungi and natural mycotoxin contamination because of their field experience of more than 20yrs as farmers. Cultural influences may therefore determine the acquisition of the intuition to identify and deal with natural mycotoxin contamination in stored foods.

There was a preponderance of male over female respondents in this present study. Presumably, women carry out duties related to food processing and cooking in addition to other ancillary activities at home and so makes them relatively busy. Indeed, Jere et al.^105^ reported that in Malawi, women generally carry out food processing and cooking activities in addition to other household chores which keep them occupied for the best part of the day. Jere et al.^105^ carried out studies related to the exposure of school children to aflatoxins (from *Aspergillus flavus*) and fumonisins (*Fusarium* species) in Malawi and confirmed the detection of high concentrations of mycotoxins in food prepared by women in addition to their other household chores. The same was true in Tanzania^106^ and Nigeria^107^. They surmised that educational level plays a major role in the detection and corrective interventions in mycotoxicosis as earlier endorsed by^108^.

Our current study showed that in Ghana, female respondents were significantly (p < 0.05) less knowledgeable than their male counterparts (data not shown), while Matumba et al.^6^ stated that women played a significant role in the hawking, preparation, and utilization of foods in Malawi. In other instances in Tanzania, Magembe et al.^97^ found that female respondents showed greater and adequate awareness of mould infection than men. Presumably, both cultural and educational differences in Africa can influence the detection of fungi, natural mycotoxin contamination, and response to practical control of storage diseases of fungal origin by their ability to identify and infer their presence in foods. In this present study, the majority of our respondents have attained tertiary education and had adequate knowledge about the presence of fungi in food while the rest had either no education background or are semi-literates with a low level of formal education. Lesuuda et al.^109^ also reported that in Kenya, the majority of their respondents were primary school leavers, and their knowledge of mycotoxin contamination of staple food and dehydrated cereal grains was significantly low (p < 0.05).

The statistical analysis managed the inequitable return of questionnaires from the regions since data for the entire country was bulked to exceed the minimum number of respondents required for significance. The majority of the respondents were familiar with the terms mould and yeasts and mycotoxins, although the majority attributed ingestion of mycotoxins to stomach ache and diarrhea while about 9% attributed mycotoxins as the agent of cancer and 15% believed mycotoxins cause immune suppressions (Table 7). Curiously, 30.8% attributed mycotoxin ingestion to diarrhea (Table 7). Clearly, their ignorance can be traced to their low level of education as reported by^97^ in Tanzania. Azaman et al.^110^ investigated stakeholders’ knowledge, attitude, and practices (KAP) towards aflatoxin contamination in peanut–based products and concluded that knowledge influences awareness as well as attitudes and conservation and management behavior of farmers. The adequate KAP score of 63.08 + 13.21% (Table 11) obtained in this present study agrees with the conclusion of^110^.

It is well known worldwide that aflatoxins, especially aflatoxin B1, is the most toxic mycotoxin with severe debilitating effects on humans and animals alike^12,111,112^. The majority of the respondents in this study (55.7%) (Table 3) mentioned aflatoxins as the major mycotoxin they were aware of followed by ochratoxin (16.3%) and fumonisins (16.1%); the remaining mycotoxins were less known (0.1–6.4%); while 5.79% had no idea about mycotoxins in foods. Toma^113^ and Matumba et al.^6^ also found from their studies in Ethiopia and Tanzania respectively, that aflatoxins are the best well-known mycotoxins. Studies in Kenya by Lesuuda et al.^109^ agree with our findings that the majority of their respondents knew very little about other mycotoxins other than aflatoxins. The pertinent literature contains information on the association between inadequate knowledge about aflatoxin contamination with high rates of exposure to aflatoxins^100,108,114,115^.

Fungal spores are ubiquitous and can settle on any kind of food substrate rich enough to support their growth and proliferation provided there are conducive environmental and physical conditions. Naturally, some staple foods, cereals and grains, fruits and vegetables, etc. provide a miscellany of nutrients and some better substrates for fungal growth than others^88^.

Respondents in this paper identified foods consumed in spite of visible fungal growth (Table 5). Fruits contaminated with fungi are discarded when completely infected. However, in many instances, mangoes, oranges and guavas etc. are trimmed to remove infected portions and then eaten. Unfortunately, the presence of the fungus imparts some toxic metabolites, and mycotoxins associated with fruits are mostly patulin, aflatoxin, ochratoxin, fusarial toxins, and *Alternaria* toxins^6^. Cereals and legumes are often infected by mycotoxins such as aflatoxins and fumonisins. Interestingly, respondents (35.5%), named bread as the most consumed food even with visible fungi and the least (0.2–0.3%) root tubers and *banku* (made from fermented maize flour). Respondents also scored 1.1% for *kenkey* (another fermented maize product) which is a popular meal in Ghana. The fermented dough for the preparation of *kenkey* made from maize grains is first soaked in water for 2–3 days, after which the floating damaged and infected grains are removed by decanting from the surface of the water. This process is followed by blending the soaked grains in a disc attrition device corn miller, and the resulting dumpling is fermented for 2–3 days before using the dumpling to prepare *kenkey*. It is known that the clearing and fermentation stages reduce the risk of mycotoxin contamination. It is not surprising that only 1.1% of the respondents indicated that maize is consumed even if visibly mouldy. Indeed, there has been only one record of aflatoxin contamination of *kenkey* in Ghana^60^ but the infection of grains and cereals and legumes in storage in silos and home barns is well documented by many workers in Ghana^7,44,58,59,64,70–73,84^.

Respondents to our structured questionnaires were aware of the health problems associated with the consumption of food contaminated with fungal toxins and named ailments such as stomach ache, diarrhea, suppression of the immune system, cancer, etc. (Table 7). This finding is at variance with the report of Matumba et al.^6^ in Malawi where consumption of mouldy foods was attributed to malaria and high fever. Lack of adequate information and minimal educational background could be the cause of such ignorance. Furthermore, the problem is accentuated by some health professionals and agricultural extension officers knowing little or not having heard about the debilitating effect of continuous ingestion of foods laden with fungal contaminants in Ghana^116,117^. On the contrary, Llesanmi and Llesanmi^118^ reported that in Nigeria, health workers were abreast with the knowledge of infection of foods by mycotoxins produced by fungi and the health risks associated with it. However, the prescribed method of removing of mouldy grains from farm produce is not as strictly adhered to. Jolly et al.^108^ stated that although farmers in some African countries are aware of the detrimental consequences of eating contaminated foods, their origin, and remedial traditional practices, this has not been strictly adhered to in practical terms. It is obvious that public education is lacking in Ghana on the occurrence of aero-mycoflora in storage facilities and in the fresh before harvest and the subsequent formation of metabolites in foods for human consumption. Cutting-edge technological methods are available for precluding fungi in storage and during food processing. Emphasis should be on adapting and applying good storage management practices and changing the behavioral lassitude to protect oneself from ingesting mycotoxin-contaminated foods. Unfortunately, the mould contaminated cereals, legumes, fruits, vegetables, etc. have been used inadvertently for animal and human feed, especially for chickens, ruminants, and others^119,120^. This is subsequently passed on in the meat produced and is unintentionally ingested with the toxin.

Kortei et al.^121^ reported that in Ghana cereals (e.g. Sorghum) with visible mouldiness have been used to prepare local alcoholic beverages with impunity leading to the consumption of mycotoxins (aflatoxins, fumonisins, ochratoxins, penicillinic acid, etc.). This viewpoint is endorsed by the data in Tables 4, 7, 11 and 12 showing the paucity of relevant information to the public on the potential hazards of mycotoxicosis.

Essentially, a “School Feeding Programme” from UNESCO (Millenium Development Goals) (UNESCO, 2000) has been introduced in some African countries to keep children at school and improve their erudition through nourishment. The formulation of food and times of the day the pupils are fed vary from country to country. Food analysis of the raw materials and food served to the children indicated the intake of mycotoxins (aflatoxins, ochratoxins, fumonisin, zearalenone, vomitoxin (deoxynivalenol) etc.) are beyond permissive levels^6,97,100,105,109^.

The Government of Ghana has adopted the 2nd Millenium Development Goals of the United Nations, seeking to promote education in all countries through many interventions including the introduction of Nutrition and School Feeding Programmes, Ghana School Feeding Programme (GSFP) began in 2005^122^. The aim is to achieve food security by providing public primary school students with one hot meal per day, usually procured from the farm produce of local farmers. It is one of the strategies for achieving the Millenium Development Goal 2 (MDGs) on hunger, poverty and primary education^123,124^. The GSFP is an indicator of the Comprehensive Africa Agricultural Development Programme CAADP Pillar 3, which seeks to enhance food security and reduce hunger in line with the UN Sustainable Development Goals (MDGs) on hunger, poverty, and malnutrition (Schoolfeeding.gov.gh).

Granted, the food ingredients used come from buffer stocks and farmer supply. There is a need to screen the raw materials for the presence of mycotoxins owing to the fact that we might be feeding our future leaders with foods containing mycotoxins with the potential of having a debilitating effect on the health of the youth. The urgency of disseminating information on the potential danger and how to handle it in foods and feeds should be rigorously addressed^125^ in the near future by our scientific food storage and management experts.

## Conclusion

In summary, there was adequate knowledge (63.8%) among the members of the Ghanaian populace regarding the knowledge and attitude of the occurrence of fungi and mycotoxins in foods as well as during storage. However, the respondents’ familiarity with the terms mold and yeasts (fungi) and the different types of mycotoxins was low. This undeniably calls for the intensification of education of the Ghanaian populace on yeasts and molds as well as mycotoxins in relation to their potential to cause grave harm to humans and animals as they occur in our foods.

## Data Availability

The datasets used and/or analyzed during the current study are available from the corresponding author upon a reasonable request.
